# The Relationship Between Heart Rate and Left Ventricular Isovolumic Relaxation During Normoxia and Hypoxia-Asphyxia in Newborn Piglets

**DOI:** 10.3389/fphys.2019.00525

**Published:** 2019-05-07

**Authors:** Wei Shen, Xin Xu, Tze-Fun Lee, Georg Schmölzer, Po-Yin Cheung

**Affiliations:** ^1^Neonatal Intensive Care Unit, Women and Children’s Hospital, Xiamen University, Xiamen, China; ^2^Neonatal Intensive Care Unit, Xiamen Children’s Hospital, Xiamen, China; ^3^Department of Pediatrics, University of Alberta, Edmonton, AB, Canada; ^4^Centre for the Studies of Asphyxia and Resuscitation, University of Alberta, Edmonton, AB, Canada

**Keywords:** hypoxia, asphyxia, newborns, heart rate, diastolic function

## Abstract

**Background:** Many asphyxiated neonates have cardiac complications including arrhythmia and contractile dysfunction. Little is known about the relationship between heart rate (HR) and diastolic function in asphyxiated neonates. We aimed to study the relationship between HR and left ventricular (LV) isovolumic relaxation (IVR) in neonates with asphyxia using a swine model.

**Methods:** Term newborn piglets were anesthetized and acutely instrumented with the placement of Millar^®^ catheter in the left ventricle. Hemodynamic parameters including HR, cardiac output, stroke volume, dP/dt_max_ and dP/dt_min_, and IVR time constant (Tau) were continuously measured and recorded. Sixteen piglets were exposed to 50-minute normocapnic hypoxia followed by asphyxia (mean of 3.2 min) by clamping of the endotracheal tube. Sham-operated piglets (*n* = 11) had no hypoxia nor asphyxia. The relationship between HR and other hemodynamic parameters were analyzed using Pearson Product Moment correlation test.

**Results:** Asphyxiated piglets had cardiogenic shock and metabolic acidosis (vs. sham-operated piglets). There were significant correlations between HR and diastolic function as shown by Tau at baseline (sham-operated: *r* = -0.68, *p* = 0.02; asphyxia: *r* = -0.55, *p* = 0.03) and during normoxia (53 min) of sham-operated piglets (*r* = -0.69, *p* = 0.02). HR and Tau was not correlated during hypoxia-asphyxia (HA) (*r* = -0.01, *p* = 0.97). Cardiac output was tightly correlated with stroke volume (*p* < 0.001) but not HR throughout the experimental period in both groups. There was no significant correlation between HR and other hemodynamic parameters during the experimental period in both groups.

**Conclusion:** We observed an uncoupling between HR and IVR Tau of the neonatal heart during HA, which deserves further studies of the relationship between HR and LV diastolic function.

## Introduction

Neonatal asphyxia is a common cause of mortality and contributes to approximately one million deaths annually worldwide (Hack and Stork, 2009). Cardiovascular dysfunction occurs in more than 60% of asphyxiated neonates and this affects organ perfusion and oxygen delivery (Weiss et al., 1976; Shah et al., 2009). Diastolic function, which is an important predictor of prognosis and mortality (Xu et al., 2008), is impaired in addition to systolic dysfunction during the ischemic insult (Jentzer et al., 2015). Often there is simultaneous tachycardia, in addition to confounding hemodynamic changes. The diastolic performance can be evaluated non-invasively by echocardiography and invasively by pressure-volume loops (PVLs) analysis including the measurement of isovolumic relaxation (IVR) time and time constant of IVR (Tau), respectively. While Schmitz et al. observed an inverse correlation between heart rate (HR) and IVR time from infancy to adolescence (Schmitz et al., 2003), De Merulis et al. (2004) observed a curvilinear relationship between HR and IVR time in healthy neonates. The relationship between diastolic function or specifically IVR time or Tau and HR during hypoxia and asphyxia is not clear. Understanding the relationship between diastolic function and HR may further help develop therapeutic strategies to improve diastolic function during hypoxia-asphyxia (HA) and may prevent adverse outcomes.

We aimed to examine the relationship between Tau, cardiac functional parameters and HR during normoxia and HA in instrumented newborn piglets. We tested the hypothesis that the correlation between HR and Tau would be disrupted during HA in newborn piglets.

## Methods

Twenty-seven mixed breed piglets (1–3 days of age, weighing 1.6 to 2.3 kg) were obtained on the day of experimentation from the University Swine Research Technology Centre. All experiments were carried out in accordance with the ARRIVE guidelines (Kilkenny et al., 2010) and recommendations of guidelines of the Canadian Council of Animal Use. The protocols were approved by the Animal Care and Use Committee (Health Sciences), University of Alberta.

### Animal Preparation

The study was performed using hemodynamic data collected in previous experiments in order to reduce animal use (Li et al., 2016; Solevåg et al., 2016). Following the induction of anesthesia using isoflurane, femoral arterial, and venous catheters (5F, Argyl^®^, Sherwood Medical Co., St. Louis, MO) were placed and positioned in the abdominal aorta and right atrium, respectively. After endotracheal intubation via tracheotomy, pressure-controlled ventilation (Sechrist infant ventilator, model IV-100; Sechrist Industries, Anaheim, CA, United States) was commenced at a respiratory rate of 16–20 breaths/min and pressure of 20/5 cmH_2_O. Oxygen saturation was kept within 90–100%, glucose level and hydration was maintained with an intravenous infusion of 5% dextrose at 10 mL/kg/h. A Millar^®^ catheter (MPVS Ultra^®^, AD Instruments, Houston, TX, United States) was inserted into the left ventricle via the left common carotid artery for continuous measurement of left ventricular (LV) pressure, composite, and segmental volumes, which were used for cardiac output calculation. During the experiment anesthesia was maintained with intravenous propofol 5–10 mg/kg/h, morphine 0.1 mg/kg/h, and additional doses of propofol (1–2 mg/kg), and morphine (0.05-0.1 mg/kg) as needed. Piglets then recovered from surgical instrumentation for 1 h when baseline hemodynamics were stable. Ventilator rate was adjusted to keep the partial arterial CO_2_ between 35–45 mmHg as determined by periodic arterial blood gas analysis. The piglet’s body temperature was maintained at 39–40°C using an overhead warmer and a heating pad.

### Hypoxia-Asphyxia Protocol

Twenty-seven piglets were block-randomized to sham-operated or HA groups. HA piglets (*n* = 16) were subjected to normocapnic alveolar hypoxia by decreasing inspired fractional oxygen concentration to 0.09-0.12 for 50 minutes followed by a period of asphyxia by clamping the endotracheal tube (mean of 3.2 minutes) to achieve severe bradycardia (25% of baseline value) and cardiogenic shock that commonly happen in asphyxiated neonates who require advanced resuscitation. Sham-operated piglets (*n* = 11) received no HA during the experimental period.

### Hemodynamic Measurements

Mean systemic arterial pressure, HR, and percutaneous oxygen saturation were continuously measured and recorded throughout the experiment with a Hewlett Packard 78833B monitor (Hewlett Packard Co., Palo Alto, CA, United States).

### Pressure-Volume Loop (PVL) Analysis

**FIGURE 1:**
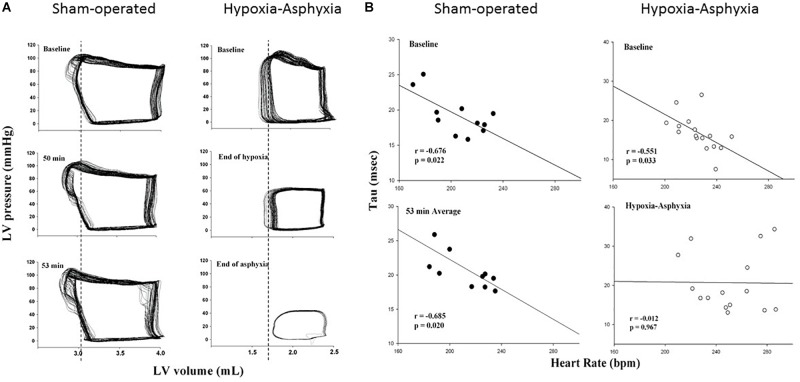
**(A)** Examples of pressure-volume loops of left ventricle (LV) measured by the Millar^®^ catheter during the experiment of a sham-operated and a hypoxia-asphyxia (HA) newborn piglet. Vertical lines, which represented the isovolumetric relaxation phase at the respective baseline of the sham-operated and HA newborn piglet, are drawn and pass through the loops of respective animals at subsequent time-points during the experimental period for comparison. **(B)** Correlation between heart rate and Tau in sham-operated and HA groups at baseline (upper panels) and during 53 minutes of normoxia or HA (lower panels).

The Millar^®^ catheter was calibrated according to the manufacturer’s instructions prior to each experiment. PVLs were volume calibrated using hypertonic saline to account for parallel conductance. Conductance catheter volumes have been shown to correlate well with cardiac MRI volumes, though they do slightly underestimate absolute volumes (Nielsen et al., 2007). All PVL data were recorded in triplicate over 10 s during an expiratory breathhold. The data acquisition rate was 1000 Hz. Examples for different phases of PVL are shown in Figure 1. Hemodynamic parameters including HR, mean/diastolic/systolic arterial pressure, stroke volume, cardiac output, ejection fraction, dP/dt_max_, dP/dt_min_, and Tau (τ) were continuously recorded using LabChart^®^ programming software (ADInstruments, Houston, TX, United States). Cardiac output was determined by thermodilution. Systolic function was evaluated using stroke volume and ejection fraction. Diastolic measures include minimum rate of ventricular pressure decline (dP/dt_min_) and Tau. While the IVR time can be evaluated by Doppler spectral analysis (Nagueh et al., 2009), Tau that based on the asymptotic analysis of PVLs measures active diastolic relaxation of LV. Tau is a commonly used measure of the lusitropic state of heart and the most established index to describe LV diastolic function. In this study we used Tau which was determined by Weiss method with the analysis of PVLs recorded.

### Data Collection and Analysis

All data were expressed as mean ± SEM. PVL analyses were performed offline using LabChart^®^ program. A minute epoch of PVL parameters prior hypoxia was used as baseline; the total HA period thereafter was used as HA changes. The analysis of PVL parameters at corresponding time periods (baseline and a 53-min average) was performed in sham-operated animals. Changes in all parameters were analyzed by two-way ANOVA. The relationship between HR, cardiac output and other hemodynamic parameters was determined using a Pearson Product Moment correlation analysis. All tests were two-sided, and *p* < 0.05 was considered significant. SigmaPlot (Systat Software Inc., San Jose, United States) was used for all statistical analysis.

## Results

The blood gasses and hemodynamic parameters of two experimental groups during the experiment are shown in Supplementary Table 1. There was hypoxemia and hypercapnia at the end of HA (PaO_2_: 22 ± 2 vs. 63 ± 2 mmHg; PaCO_2_: 77 ± 5 vs. 37 ± 2 mmHg of the sham-operated group, respectively, both *p* < 0.05). After asphyxiation, HR was significantly lowered, compared with the sham-operated group (69 ± 8 vs. 207 ± 10 bpm, respectively). Mean arterial pressure and cardiac output also significantly decreased (24 ± 4 vs. 71 ± 4 mmHg and 66 ± 13 vs. 194 ± 23 mL/kg/min in the sham-operated group, respectively). The HA piglets developed severe acidosis and hyperlactatemia (arterial pH: 6.82 ± 0.03 vs. 7.39 ± 0.02; base deficit: 22 ± 1 vs. 2 ± 1 mmol/L; plasma lactate level: 14.1 ± 0.3 mM vs. 4.4 ± 0.6 mM of sham-operated piglets, respectively, both *p* < 0.05).

As shown in the Figure 1, HR significantly correlated with Tau at baseline in both groups (sham-operated group: *r* = -0.68, *p* = 0.02; HA group: *r* = -0.55, *p* = 0.03) and this negative correlation persisted throughout the experimental period for the sham-operated group (*r* = -0.69, *p* = 0.02). The relationship between HR and Tau changed and were not correlated during HA (*r* = -0.01, *p* = 0.97).

There were positive correlations between HR and dP/dt_max_ at baseline in both groups (sham-operated group: *r* = 0.68, *p* = 0.02; HA group: *r* = 0.45, *p* = 0.08). The relationship between HR and dP/dt_max_ was not significant in both groups during HA and the corresponding period of sham-operated group (HA group: *r* = 0.42, *p* = 0.12; sham-operated group: *r* = 0.33, *p* = 0.32). There was no significant correlation between HR and other hemodynamic parameters at baseline and throughout the experiment in both groups (*r* = —0.02∼0.42—, *p* ≥ 0.12).

The correlations between cardiac output and stroke volume were significant in both groups at baseline and remained unaffected during HA (*r* = 0.96∼0.99, all *p* < 0.001). Cardiac output did not correlate with HR, dP/dt_max_, dP/dt_min_, and Tau throughout the experimental period in both groups (all *p* > 0.05).

## Discussion

Significant relationship has been observed between HR and IVR time, suggesting the predictability of IVR time by HR (Schmitz et al., 2003; De Merulis et al., 2004). However, the relationship was observed in healthy neonatal subjects (De Merulis et al., 2004) but not in pathological conditions including HA. During HA there are associated changes in the hemodynamic factors that may confound the relationship between IVR time or Tau and HR. In this study, we confirmed the significant correlation between HR and Tau and the disruption of this correlation during HA. The disrupted relationship and thus predictability of Tau by HR may be a direct effect and the associated confounding hemodynamic changes.

Tau has been shown to be prolonged in adults with heart failure and a normal ejection fraction (Zile et al., 2004). In healthy adults, the upper limit of Tau values is 48 ms, however, there is a lack of established normative data for Tau in neonates. It remains controversial to apply adult normal values in neonates with asphyxia. Furthermore, Tau value in children with single ventricle physiology was lower compared to that in adults (Chowdhury et al., 2014). In our study, the mean Tau value at baseline ranged from 17 to 20 ms and the asphyxia group had a mean Tau of 57 ms at the end of HA.

Diastolic dysfunction is a hemodynamic hallmark of heart failure. Many reports attempt to demonstrate the importance of early recognition of cardiac dysfunction by using echocardiography or other methods (Feng et al., 2016; Jang et al., 2016). While systolic function could be evaluated using echocardiography (such as ejection fraction, shortening fraction), the assessment of diastolic dysfunction (e.g., IVR time) by non-invasive method is challenging (Nagueh et al., 2009; Savage et al., 2010). Invasive cardiac catheterization can provide insight into diastolic function (Tau). Tau is a standard measurement of active diastolic relaxation using the analysis of conductance-derived PVLs. Nevertheless, animal and human studies have confirmed that the propagation velocity of early flow into the LV cavity measured by color M-mode Doppler is closely related to Tau (Stugaard et al., 1993). Further in patients with single ventricular physiology, Chowdhury et al. (2014) reported that diastolic function (Doppler E:A, lateral E:E’, and IVR time) measured by echocardiography significantly correlated with that by PVLs (*r* = 0.75-0.83). Interestingly, ventricular PVL could also be obtained by 3D real-time echocardiography and mini-pressure wire in neonates with congenital heart disease (Herberg et al., 2013).

Approximately 60% asphyxiated neonates have cardiovascular symptoms including arrhythmia, systolic and diastolic dysfunction (Shah et al., 2009). Abnormalities of diastolic function are recognized as an important determinant of heart failure symptoms in the context of normal and abnormal systolic function. Ventricular diastolic dysfunction was present in more than half of the neonates with either moderate or severe asphyxia and patients with severe asphyxia had higher grade of diastolic dysfunction (Shahidi et al., 2017). Although LV systolic function was preserved, diastolic function was impaired in hypoxia, such diastolic dysfunction may be caused by functional and anatomical ventricular interaction associated with pulmonary hypertension and right ventricular hypertrophy (Itoh et al., 2009).

The relationship between HR and diastolic function has been studied using echocardiography with variable results (Brun et al., 1992; Burns et al., 2007). Of note, HR is the major determinant of diastolic filling pattern during growth in childhood (Arsos et al., 2002). It is therefore interesting to study the relationship between HR and Tau in neonates during HA. While the inverse relationship during normoxia is consistent with that in healthy adults (Savage et al., 2010), the correlation became not significant during the period of HA, suggesting an uncoupling or disrupted relationship between HR and Tau with an impaired force-frequency relationship.

### Limitations

In addition to the limitations related to animal model of neonatal asphyxia, our study did not control other factors that may also affect diastolic function measured by Tau using PVLs. These factors include preload, afterload and myocardial oxygen consumption, which are altered during HA. While Millar^®^ PVL software calculates Tau using one asymptotic model, it is important to confirm our findings using Tau calculated by other models. The study design and preliminary nature of our findings also precluded us from investigating the pathogenetic mechanisms. Further investigations using isolated heart perfusion technique may help understand the relationship and mechanisms between HR and Tau.

Volatile anesthetic agents including isoflurane used in this study for induction, might have some detrimental effects on LV function (Manohar and Parks, 1984). Similar doses and durations of anesthesia were used for both experimental groups. Small sample size and collation of data from two sets of experiments further limit the strength of our conclusion.

## Conclusion

We demonstrated that HR and Tau coupled significantly at normoxia but uncoupled during HA in a swine model of neonatal asphyxia. Further studies are required to understand the mechanism of the relationship in the neonatal heart during asphyxia.

## Ethics Statement

This study was carried out in accordance with the recommendations and guidelines of Canadian Council of Animal Care. The protocol was approved by the Animal Care and Use Committee, University of Alberta.

## Author Contributions

P-YC, T-FL, GS, WS, and XX contributed conception and design of the study. WS, XX, and T-FL organized the database. WS and TFL performed the statistical analysis. P-YC, T-FL, GS, WS, and XX performed data interpretation; WS wrote the first draft of the manuscript. All authors contributed to manuscript revision, read and approved the submitted version.

## Conflict of Interest Statement

The authors declare that the research was conducted in the absence of any commercial or financial relationships that could be construed as a potential conflict of interest.
