# Supplementary material for: The Relationship Between Heart Rate and Left Ventricular Isovolumic Relaxation During Normoxia and Hypoxia-Asphyxia in Newborn Piglets
Source: Front Physiol. 2019 May 7;10:525. doi: 10.3389/fphys.2019.00525 (PMC6514222; doi:10.3389/fphys.2019.00525)
Supplement: Supplementary file 1 [file Table_1.DOC]

**Table** Hemodynamic parameters and arterial blood gases of newborn piglets in sham-operated and Hypoxia-Asphyxia groups.

*p<0.05 vs. sham-operated group at the same time point and ^#^p<0.05 vs. baseline of the same experimental group (Two-way ANOVA).

|  | Sham-operated (n=11) | | Hypoxia-Asphyxia (n=16) | |
| --- | --- | --- | --- | --- |
|  | Baseline | End | Baseline | End |
| Heart Rate (bpm) | 203±7 | 210±9 | 221±5* | 69±8*^,#^ |
| Mean Blood Pressure (mmHg) | 73±3 | 71±4 | 73±3 | 24±4*^,#^ |
| Cardiac Output (mL/kg/min) | 208±25 | 194±23 | 243±33 | 66±13*^,#^ |
| Tau (msec) | 20±1 | 21±2 | 17±1* | 57±9*^,#^ |
| Stroke Volume (mL/kg) | 0.9±0.1 | 0.8±0.2 | 1.1±0.1 | 0.5±0.1^#^ |
| Ejection Fraction (%) | 21±2 | 22±3 | 27±2* | 13±2*^,#^ |
| End Diastolic Pressure (mmHg) | 8±1 | 9±2 | 8±1 | 15±2*,# |
| End Diastolic Volume (mL/kg) | 4.3±0.3 | 3.9±0.4 | 4.4±0.4 | 4.2±0.5 |
| dp/dt max (mmHg/s) | 4048±318 | 3758±380 | 4824±244* | 1097±131*^,#^ |
| dp/dt min (mmHg/s) | -3925±423 | -4115±370 | -4146±253 | -1112±142*^,#^ |
|  |  |  |  |  |
| Arterial pH | 7.39±0.01 | 7.39±0.02 | 7.38±0.01 | 6.82±0.03*^,#^ |
| PaO_2_ (mmHg) | 61±2 | 63±2 | 60±2 | 22±2*^,#^ |
| PaCO_2_ (mmHg) | 40±1 | 37±2 | 40±2 | 77±5*^,#^ |
| BE (mmol/L) | -0.7±1 | -2.3±1 | -2.3±1 | -22±1*^,#^ |
| HCO_3_ (mmol/L) | 24.1±0.7 | 23.0±0.6 | 23.0±0.7 | 12.2±0.5*^,#^ |
| Lactate (mmol/L) | 4.1±0.5 | 4.4±0.6 | 3.6±0.3 | 14.1±0.3*^,#^ |
| Hemoglobin (g/L) | 76±4 | 79±3 | 80±4 | 87±3 |
